# Ultrasonography of Leprosy Neuropathy: A Longitudinal Prospective Study

**DOI:** 10.1371/journal.pntd.0005111

**Published:** 2016-11-16

**Authors:** Helena Barbosa Lugão, Marco Andrey Cipriani Frade, Wilson Marques-Jr, Norma Tiraboschi Foss, Marcello Henrique Nogueira-Barbosa

**Affiliations:** 1 Dermatology Division, Department of Internal Medicine, Ribeirão Preto Medical School, University of São Paulo, Ribeirão Preto, São Paulo, Brazil; 2 Department of Neurology, Ribeirão Preto Medical School, University of São Paulo, Ribeirão Preto, São Paulo, Brazil; 3 Radiology Division, Department of Internal Medicine, Ribeirão Preto Medical School, University of São Paulo Ribeirão Preto, São Paulo, Brazil; University of Tennessee, UNITED STATES

## Abstract

**Background:**

Previous studies have shown that leprosy multi-drug therapy (MDT) does not stop the progression of nerve function impairment. There are no prospective studies investigating the evolution of nerve anatomic abnormalities after treatment. We examined leprosy patients aiming to investigate the evolution of nerve ultrasonography (US) abnormalities and the risk factors for poor outcomes after MDT.

**Methodology/Principal findings:**

We performed bilateral US of the ulnar (U), median (M) and common fibular (CF) nerves in 9 paucibacillary (PB) and 64 multibacillary (MB) patients before and after MDT. Forty-two patients had leprosy reactions (type 1, type 2, acute neuritis) during the study. We analyzed nerve maximum cross-sectional areas (CSA), echogenicity and Doppler signal. Poor outcomes included a post-treatment CSA above normal limits with a reduction of less than 30% (U, M) or 40% (CF) from the baseline, echogenicity abnormalities or intraneural Doppler in the post-treatment study. We found that PB and patients without reactions showed significant increases in CSA at CF, whereas MB and patients with reactions had CSA reduction in some nerves after treatment (p<0.05). Despite this reduction, we observed a greater frequency of poor CSA outcomes in the MB compared to the PB (77.8% and 40.6%; p>0.05) and in the patients with reactions compared to those without (66.7% and 38.7%; p<0.05). There was significantly higher odds ratio (7.75; 95%CI: 1.56–38.45) for poor CSA outcomes only for M nerve in patients with reactions. Poor echogenicity outcomes were more frequent in MB (59.4%) compared to PB (22.2%) (p<0.05). There was significant association between poor Doppler outcomes and neuritis. Gender, disease duration, and leprosy classification were not significant risk factors for poor outcomes in CSA, echogenicity or Doppler.

**Conclusions/Significance:**

US nerve abnormalities can worsen after treatment despite the leprosy classification or the presence of reactions.

## Author Summary

Leprosy can lead to functional and anatomical changes in the peripheral nerves. Previous studies have shown that the anti-bacterial treatment cannot stop the progression of nerve function abnormalities; to our knowledge, there are no previous prospective studies investigating the evolution of nerve anatomical abnormalities. Seventy-three leprosy patients underwent bilateral ultrasonography of the ulnar, median and common fibular nerves before and after treatment. We analyzed thickening and asymmetry measurements, nerve architecture (echogenicity abnormalities) and vascularization (Doppler signal). We observed worsening of thickening in the common fibular nerve in the paucibacillary and in the patients without reactions. On the other hand, we observed reduction in the thickening and asymmetry measurements for some nerves in the multibacillary and in the patients with reactions. Although we observed reduction in the measurements of these two groups, they presented higher frequencies of echogenicity abnormalities, indicating that these reductions might have been due to evolution to nerve fibrosis and atrophy. The frequencies of Doppler signal were similar between the pauci and multibacillary patients. We observed association between Doppler signal and the presence of acute neuritis. Our results indicate that the nerve anatomic abnormalities assessed by ultrasonography can worsen after treatment despite the patient classification and the presence of reactions.

## Introduction

Leprosy is the leading infectious cause of disability [1,2]. Neurological involvement may start before diagnosis or either during or after treatment, leading to functional impairments and deformities [1,3–5].

Nerve palpation can detect thickening, but it is examiner-dependent and it demands practical training [6]. One study that evaluated the reliability of nerve palpation detected poor agreement between trained staff [7]. Recently, ultrasonography (US) has been used to document anatomical nerve abnormalities in patients with leprosy [8–15]. US provides objective measurements of nerve enlargement and asymmetry [12,15] and can identify more extensive involvement than clinical examination [9]. Additionally, leprosy patients can have nerve enlargement detected with US without functional impairment identified in neurophysiological studies and vice versa [10,16].

There are prospective studies investigating functional impairment during and after multi-drug treatment (MDT) [17–19]; however, to our knowledge, there are no longitudinal studies investigating the evolution of nerve ultrasonographic abnormalities after MDT in leprosy patients. The purpose of this study was to investigate the evolution of US abnormalities in leprosy patients and the risk factors for poor outcomes after treatment. We hypothesize that nerve abnormalities detected by US may not show regression after treatment.

## Methods

### Ethics statement

The study was conducted at the Leprosy Reference Center of the Ribeirão Preto Medical School Hospital—University of São Paulo (HCFMRP-USP). The Ethics Committee of the HCFMRP-USP approved the study (process n°02663112.0.0000.5440). Written informed consent was obtained from all participants. The parents provided written consent on behalf of the minor participants.

### Subjects

**Fig 1:**
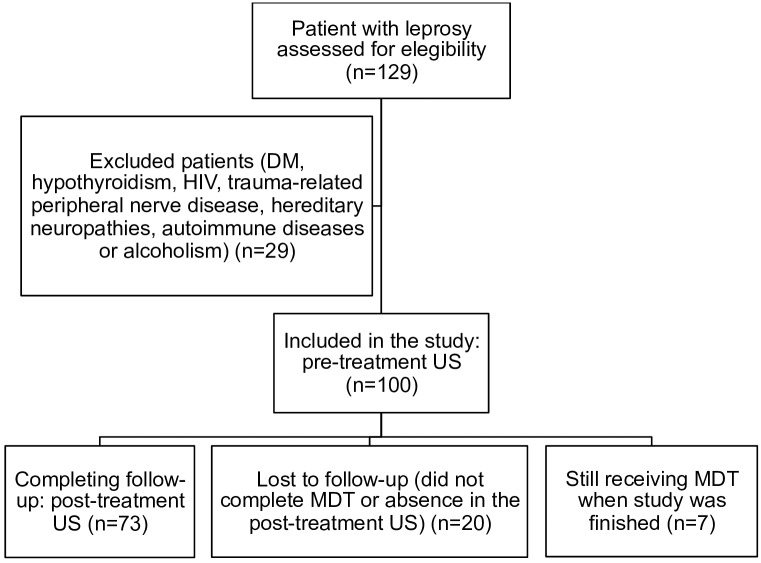
Patient flowchart reporting numbers of individuals at each stage of study. Legend: DM: diabetes mellitus; HIV: human immunodeficiency virus; US: peripheral nerve ultrasonography; MDT: multi-drug therapy.

The present paper shows the results of the prospective US evaluation after treatment. The patient flowchart reporting numbers of individuals at each stage of the study is shown in Fig 1. One hundred patients underwent bilateral high-resolution US of the peripheral nerves before starting the World Health Organization (WHO) MDT. The pre-treatment results have been published [15]. Seventy-three leprosy patients repeated US after completion of MDT (31 women and 42 men, age range 8–86 years, age mean 45.3±17.3). To reduce bias, patients underwent post-treatment US approximately 2 years after the pre-treatment exam, regardless of their classification. The group of paucibacillary patients (PB) underwent post-treatment US an average of 27.6 months after the pre-treatment exam, and the multibacillary patients (MB) underwent post-treatment US an average of 21.8 months afterwards (p>0.05).

Leprosy diagnosis was established based on clinical signs and symptoms, skin smears, skin biopsy, and neurophysiological examination when necessary. Patients were classified into the following five groups according to the Ridley-Jopling classification [20]: tuberculoid (TT), borderline-tuberculoid (BT), borderline-borderline (BB), borderline-lepromatous (BL), and lepromatous (LL). Patients with the indeterminate form (I) of leprosy were also included. Cases of I and TT leprosy were classified as PB, whereas the other forms were classified as MB according to the WHO operational classification [21].

The patients’ medical charts were reviewed for the identification of leprosy reactions. Type 1 cutaneous reactions were defined as the presence of erythema and edema of skin lesions associated or not with new lesions. There may be accompanying neuritis and edema of the hands, feet, or face. Type 2 cutaneous reactions (erythema nodosum leprosum) were defined as the presence of tender subcutaneous skin lesions. There may be accompanying neuritis, iritis, arthritis, orchitis, dactylitis, lymphadenopathy, edema, and fever. Neuritis was diagnosed if the patient presented with acute swelling and/or functional impairment of peripheral nerves with spontaneous pain or tenderness on palpation. Anti-reaction treatment was started as soon as the reaction was detected. Type 1 reactions and neuritis were treated with corticosteroids (initial dose 0.5 to 1.0mg/kg/day) for at least 16 weeks. Type 2 reactions were treated with thalidomide (100 to 300mg/day) and/or corticosteroids. Two patients with severe and recalcitrant neuritis received azathioprine associated with corticosteroids. For the statistical analysis of quantitative variables (CSA, ΔCSA and ΔUtpt) patients were classified according to the presence of any type of leprosy reaction or absence of reactions during the study. We analyzed the results dividing the patients in these two groups because the presence of reactions, independently of its type, could lead to additional nerve inflammation. Besides, as patients can have concomitant reactions (for example, neuritis and type 1 cutaneous reaction), the separated analysis of reactions could lead to bias.

Patients were also classified according to the disease duration, which was defined as the interval between the first symptoms and the realization of the pre-treatment US. The disease duration was considered short (less than 2 years), moderate (between 2 and 5 years) or long (more than 5 years).

The control group included 41 healthy volunteers (27 women and 14 men, age range 12–80 years, mean age 37±17.4 years) who were submitted to the same US exam protocol as the leprosy patients. The results of the control group were used to calculate the upper limits for the nerve cross-sectional areas (CSAs) (mean plus 2 standard deviations).

Leprosy patients with diabetes mellitus, hypothyroidism, human immunodeficiency virus infection, trauma-related peripheral nerve disease, hereditary neuropathies, autoimmune diseases or alcoholism were not included in the study. The control group comprised healthy volunteers without household contact with leprosy patients and without other potential causes of peripheral neuropathies (diabetes mellitus, hypothyroidism, human immunodeficiency virus infection, trauma-related peripheral nerve disease, hereditary neuropathies, autoimmune diseases, alcoholism).

### Ultrasonography

The US exam was performed as previously described [15]. Musculoskeletal radiologists with previous fellowship training in nerve imaging performed all US sessions using a 12-MHz linear transducer model HDI-11 (Philips Medical Systems, Bothell, Washington, USA). The ulnar (at the cubital tunnel area—Ut—and proximal to the tunnel—Upt), median (M) and common fibular (CF) nerves were systematically scanned along the transverse and longitudinal axes. Ulnar nerves were scanned from the middle third of the arm to the middle third of the forearm. M nerves were evaluated at the middle and distal thirds of the forearm. CF nerves were evaluated from the distal third of the thigh to the knee at the fibular head. In some cases, it was not possible to examine nerves bilaterally due to amputation, cutaneous ulcers or other cutaneous alterations at the site of examination. Nerve CSAs were measured by freehand delimitation at the inner borders of the echogenic rims of the nerves at the level of maximum thickening.

The CSA measurements were used to calculate asymmetry. These following measurements were determined: (1) CSA index (ΔCSA), absolute difference between CSAs for each nerve point from one side to the contralateral side; (2) Ut-Upt index (ΔUtpt) of the ulnar nerve, absolute difference between the largest and smallest CSAs of the Upt and Ut points of the ulnar nerves on the same side.

The color Doppler settings were chosen to optimize the identification of weak signals from vessels with a slow velocity and to avoid artifacts. To increase the vascular depiction, the power Doppler mode was used with a PRF of 0.7 to 1 kHz. The detection of intraneural or epineural Doppler signal was considered indicative of nerve hypervascularity and therefore an abnormal finding.

The nerve echogenicity was also classified as normal or abnormal. Nerves were classified as abnormal if they showed hypoechoic or hyperechoic areas or focal thickening with loss of the normal fascicular pattern.

### Inter-observer reliability

A pilot study with 15 leprosy patients and 5 healthy volunteers was performed to evaluate the inter-observer reliability of US. Two radiologists, who were blinded to the patient diagnosis and to the measurements of the other radiologist, performed consecutive CSA measurements. The 90th percentile of the inter-observer variation ranged between 33.3–35.6% at Upt, 31.4–37.9% at Ut, 25.6–30.9% at M, and 35.4–45.2% at the CF nerve. The intraclass correlation coefficient (ICC) was above 0.77 for all nerves examined, which is considered a strong inter-observer agreement. ICC was classified as follows: poor (0–0.2), fair (0.3–0.4), moderate (0.5–0.6), strong (0.7–0.8), and almost perfect (>0.8) [22].

### Criteria for poor outcomes on ultrasonography

Considering the 90th percentile of the inter-observer variation, we defined a significant change in the nerve CSA between pre- and post-treatment exams as a >30% difference from baseline for the Upt, Ut and M nerves and >40% difference for the CF nerve.

Poor CSA outcomes were defined as a post-treatment CSA above the normal limits (mean plus 2 standard deviations of the control group measurements) and with less than a 30% (Upt, Ut, M) or 40% (CF) reduction from the baseline.

Poor echogenicity outcome was defined as the presence of echogenicity abnormalities in at least one nerve in the post-treatment study.

Poor Doppler outcome was defined as the detection of intraneural or perineural Doppler signal in at least one nerve in the post-treatment study.

### Statistical analysis

Statistical analysis was performed using SAS software version 9.0 (SAS Institute Inc., Cary, NC). We performed linear regression controlling for the effects of confounding factors (operational classification, reactions, disease duration, and gender). The logistic regression was used to estimate the odds ratio (OR) and we considered the following risk factors for poor outcomes: operational classification, reactions, disease duration, and gender. The statistical analysis also included Chi-square and McNemar tests. Probability (p) values less than 0.05 were considered significant.

## Results

The clinical classifications and incidences of reactions are presented in Table 1. Some patients presented with cutaneous reactions (types 1 or 2) associated with neuritis. For clinical data and pre- and post-treatment US findings of each patient see supporting information (S1 Table).

**Table 1:** Clinical data for the patients included in the study.

Leprosy classification				Leprosy reactions			
WHO*	n (%)	RJ**	n (%)	Neuritis	Type 1	Type 2	No reactions
PB	9 (12.3%)	I	3 (4.1%)	0	0	0	3
		TT	6 (8.2%)	3	1	0	2
MB	64 (87.7%)	BT	21 (28.8%)	9	3	0	10
		BB	29 (39.7%)	10	8	0	13
		BL	10 (13.7%)	3	6	1	2
		LL	4 (5.5%)	1	0	3	1
Total	73		73	42			31

n: number of patients, with percentages in parentheses.

*WHO: Operational classification proposed by the World Health Organization.

** RJ: Modified Ridley-Jopling classification.

The prospective analyses of CSA measurements for the PB and MB patients are shown in Table 2. No significant differences between pre and post-treatment asymmetry measurements (ΔCSA and ΔUtpt) were observed in the PB and MB.

**Table 2:** CSA results before and after treatment in PB and MB patients.

		PB (9 patients)			MB (64 patients)		
	Nerves	Pre-treatment CSA (mm^2^)	Post-treatment CSA (mm^2^)	p-value	Pre-treatment CSA (mm^2^)	Post-treatment CSA (mm^2^)	p-value
**Right side**	**Ulnar (Upt)**						
	mean±SD	6.8±1.6	6.4±1.2	0.50	8.5±4.9	9.0±5.9	0.60
	median	6.9	6.7		7.1	7.0	
	**Ulnar (Ut)**						
	mean±SD	6.9±2.8	6.4±1.6	0.78	11.1±6.1	9.6±4.9	0.04*
	median	6.0	7.0		9.0	8.7	
	**Median**						
	mean±SD	6.1±1.6	6.0±1.8	0.77	8.9±4.9	9.2±4.7	0.98
	median	6.3	6.5		7.3	7.4	
	**Common fibular**						
	mean±SD	10.0±5.4	14.0±5.6	0.07	17.3±8.7	17.2±7.5	0.84
	median	9.6	14.0		14.1	14.2	
**Left side**	**Ulnar (Upt)**						
	mean±SD	7.5±2.4	6.2±1.7	0.06	11.0±10.1	10.2±8.0	0.03*
	median	6.1	6.5		8.0	7.0	
	**Ulnar (Ut)**						
	mean±SD	6.7±1.4	6.8±1.1	0.93	12.5±14.3	10.3±7.1	0.02*
	median	7.0	7.1		8.5	8.5	
	**Median**						
	mean±SD	6.3±0.9	5.9±1.0	0.54	9.8±5.7	9.3±4.7	0.04*
	median	6.4	5.9		8.0	8.0	
	**Common fibular**						
	mean±SD	17.4±18.6	24.1±21.3	<0.01*	17.8±9.7	17.2±7.2	0.30
	median	10.6	16.8		15.0	16.0	

CSA: cross-sectional area; SD: standard deviation; PB: paucibacillary patients; MB: multibacillary patients; Upt: ulnar nerve, proximal to the cubital tunnel; Ut: ulnar nerve at the cubital tunnel;

*: statistically significant.

Linear regression.

The majority (77.8%) of MB patients had at least one nerve with a poor CSA outcome compared to 40.6% of the PB (p>0.05). The analysis of each nerve revealed that none of the PB had poor CSA outcomes for the Upt, Ut, and M nerves. The frequency of poor CSA outcomes in the MB for the right and left side nerves were 22.6% and 27.9% for the Upt, 25% and 20.6% for the Ut, and 25% and 27.4% for the M nerve, respectively. For the CF nerve, we observed similar frequencies of poor CSA outcomes in the PB (11.1% right and 22.2% left) and MB patients (20.3% right and 17.4% left). The OR revealed similar risks for poor CSA outcomes between PB and MB at the CF nerve. For the other nerves it was not possible to calculate the OR because none PB patient had poor CSA outcomes.

The pre- and post-treatment CSA, ΔCSA and ΔUtpt measurements for the groups with and without reactions are shown in Tables 3 and 4.

**Table 3:** CSA results before and after treatment in patients without and with reactions.

		No reactions (31 patients)			Reactions (42 patients)		
	Nerves	Pre-treatment CSA (mm^2^)	Post-treatment CSA (mm^2^)	p-value	Pre-treatment CSA (mm^2^)	Post-treatment CSA (mm^2^)	p-value
**Right side**	**Ulnar (Upt)**						
	mean±SD	6.8±2.9	7.7±4.6	0.75	9.4±5.4	9.5±6.2	0.37
	median	6.2	6.1		8.0	7.0	
	**Ulnar (Ut)**						
	mean±SD	10.3±6.4	9.1±4.7	0.18	10.8±5.7	9.3±4.9	0.62
	median	8.0	7.0		8.8	8.0	
	**Median**						
	mean±SD	8.0±4.7	7.5±3.1	0.66	9.0±4.8	9.9±5.2	0.88
	median	7.0	7.0		7.5	7.7	
	**Common fibular**						
	mean±SD	16.3±10.1	15.8±7.0	0.36	16.5±7.6	17.6±7.6	0.12
	median	13.0	14.0		13.8	16.0	
**Left side**	**Ulnar (Upt)**						
	mean±SD	7.7±3.4	7.2±3.0	0.08	12.9±11.9	11.7±9.3	0.03*
	median	7.4	6.9		8.1	7.9	
	**Ulnar (Ut)**						
	mean±SD	9.7±5.9	9.3±5.0	0.80	13.3±16.9	10.3±7.8	0.16
	median	7.9	7.9		8.3	8.0	
	**Median**						
	mean±SD	8.8±5.0	8.0±3.8	0.26	9.9±5.8	9.6±4.9	0.29
	median	7.4	7.0		7.0	7.9	
	**Common fibular**						
	mean±SD	15.8±8.1	16.6±6.5	0.02*	19.3±12.6	19.2±12.0	0.16
	median	13.8	16.0		14.0	16.7	

CSA: cross-sectional area; SD: standard deviation; Upt: ulnar nerve, proximal to the cubital tunnel; Ut: ulnar nerve at the cubital tunnel;

*: statistically significant.

Linear regression.

**Table 4:** Asymmetry measurements (ΔCSA and ΔUtpt) before and after treatment in patients without and with reactions.

		No reactions (31 patients)			Reactions (42 patients)		
Variable	Nerves	Pre-treatment	Post-treatment	p-value	Pre-treatment	Post-treatment	p-value
**ΔCSA (mm**^**2**^**)**	**Ulnar (Upt)**						
	mean±SD	1.6±1.6	2.0±3.5	0.91	5.2±9.2	4.3±6.9	0.20
	median	1.1	1.1		1.5	1.9	
	**Ulnar (Ut)**						
	mean±SD	2.7±4.9	2.2±2.6	0.44	6.2±15.0	4.2±7.2	0.04*
	median	1.2	1.2		2.0	2.0	
	**Median**						
	mean±SD	1.8±2.6	1.8±1.7	0.68	2.3±3.8	3.1±4.6	0.74
	median	1.0	1.6		1.0	1.2	
	**Common fibular**						
	mean±SD	5.9±8.3	4.0±4.2	0.43	4.4±8.5	4.6±9.8	0.10
	median	3.0	2.8		2.0	3.0	
**ΔUtpt (mm**^**2**^**)**	**Right ulnar nerve**						
	mean±SD	4.2±5.9	3.2±3.4	0.85	3.2±2.9	2.7±3.0	0.90
	median	2.5	2.0		2.0	2.0	
	**Left ulnar nerve**						
	mean±SD	3.8±5.0	3.1±3.5	0.50	5.2±8.1	4.6±7.1	0.04*
	median	2.0	2.0		2.6	2.0	

ΔCSA: differential CSA (cross-sectional area) index; ΔUtpt: differential ulnar pre-tunnel and tunnel index; SD: standard deviation; Upt: ulnar nerve, proximal to the cubital tunnel; Ut: ulnar nerve at the cubital tunnel;

*: statistically significant.

Linear regression.

We observed higher frequencies of poor CSA outcomes in at least one nerve in patients with reactions (66.7%) compared to patients without reactions (38.7%) (p<0.05). The results for each nerve are shown in Table 5.

**Table 5:** Frequency of poor CSA outcomes after treatment in patients without and with reactions.

Nerve	Frequency of CSA poor outcomes		OR	CI95%	p-value
	No reactions	Reactions			
	(31 patients)	(42 patients)			
**Ulnar (Upt)**					
Right	16.1%	22.5%	1.21	0.34–4.27	0.77
Left	13.3%	32.5%	2.78	0.78–9.95	0.12
**Ulnar (Ut)**					
Right	22.6%	21.4%	0.78	0.24–2.50	0.67
Left	20.0%	16.7%	0.76	0.22–2.64	0.66
**Median**					
Right	6.5%	34.2%	7.75	1.56–38.45	0.01*
Left	20.0%	27.5%	1.39	0.43–4.46	0.58
**Common Fibular**					
Right	12.9%	23.8%	1.80	0.47–6.84	0.39
Left	16.1%	19.5%	1.23	0.33–4.56	0.76

OR: odds ratio; CI95%: 95% confidence interval; Upt: ulnar nerve, proximal to the cubital tunnel; Ut: ulnar nerve at the cubital tunnel;

*: statistically significant.

Logistic regression.

Considering the entire group of patients (n = 73), we observed a higher frequency of echogenicity abnormalities in the post-treatment exam (54.8%) compared to the pre-treatment (42.5%) (p<0.05). Only two patients who had echogenicity abnormalities before treatment showed improvement after treatment and 11 patients who had no echogenicity abnormalities before treatment developed abnormalities in the post-treatment US. We observed a lower frequency of Doppler detection after treatment (19.2% pre-treatment and 8.3% post-treatment, p>0.05). Among the 14 patients who had Doppler signal detection in at least one nerve before treatment, only one maintained Doppler detection and 13 showed improvement. Considering the presence of each type of reaction, we observed that none of the patients with type 2 cutaneous reactions presented intraneural Doppler detection both on pre-and post-treatment exams. Six patients had Doppler signal detection after treatment: 2 patients had type 1 reaction associated with neuritis, 3 patients had neuritis without cutaneous reactions, and 1 patient had no clinical signs leprosy reactions during the post-treatment US and evolved with neuritis a couple of months later.

We observed higher frequencies of poor echogenicity outcomes in MB (59.4%) compared to PB (22.2%) (p<0.05), without a significant increment in the OR (OR: 4.42; CI95%: 0.81–24.24; p>0.05). 9.4% of the MB had poor Doppler outcome and none of the PB patients showed Doppler detection after treatment (p>0.05), precluding the estimation of the OR.

Patients with reactions presented higher frequencies of poor echogenicity outcomes (61.9%) compared to the patients without reactions (45.2%) (p>0.05), without increment in the OR (OR: 1.97; CI95%: 0.74–5.27; p>0.05). There was no significant association between poor echogenicity outcomes and the presence of type 1 reactions, type 2 reactions or neuritis. The frequency of poor Doppler outcomes was also non-significantly higher in the patients with reactions (11.9%) compared with those without reactions (3.2%). It was not possible to calculate the OR for this variable due to quasicomplete separation [23]. Considering each type of reaction separately, we observed significant association between Doppler detection and the presence of neuritis (p = 0.02). There was no significant association between Doppler signal and types 1 and 2 cutaneous reactions.

Gender and disease duration were not significant risk factors for poor outcomes for CSA, echogenicity or Doppler.

## Discussion

In this first prospective study investigating ultrasonographic nerve abnormalities in leprosy we found that the neural involvement may not improve after treatment, in accordance with the findings of previous clinical and electrophysiological studies [17–19,24–27]. Furthermore, US findings can worsen despite the operational classification and the presence of reactions, as demonstrated by the CSA increase in the CF nerve in PB and in patients without reactions and by the increased frequency of echogenicity abnormalities detected in the post-treatment evaluation. We expected to detect higher odds for poor outcomes in the MB and in the patients with reactions, but we observed increments in the OR only for the right median nerve CSA in patients with reactions; the other analyses of the OR (CSA, echogenicity, and Doppler) revealed similar odds between PB and MB and between patients with and without reactions. These results are very important because there are few studies that have investigated imaging findings in leprosy neuropathy, and all of them were transversal studies [8–10,12,15,16,28].

In our study, we did not investigate nerve function; we only aimed to describe anatomical nerve changes detected by US. Nevertheless, our findings can be compared to those of previous electrophysiology studies. We found that PB patients had a significant increase in the CF nerve CSA, with a pronounced difference between the pre- and post-treatment mean and median values. This nerve also presented an expressive percentage of poor CSA outcomes in the PB, with similar OR between PB and MB. The only published study that has investigated the evolution of electrophysiological findings before and after treatment in PB and MB (15 and 17 patients, respectively) [19] found that 3 PB and 2 MB patients had clinical and/or electrophysiological signs of deterioration and the lower extremity nerves were more frequently and severely affected than the upper extremity nerves in both groups. These results indicate that PB patients can have deterioration of imaging and functional findings after treatment, suggesting that the neural inflammatory process may continue after healing the *M*. *leprae* infection. The CF nerve was also the most frequently enlarged nerve in PB patients before treatment [15], emphasizing the importance of the investigation of the involvement of lower extremity nerves (anatomical and/or functional).

The group of patients who did not have reactions during the study also presented significant increases in the CF nerve CSA after treatment. One previous prospective study [17] that investigated the electrophysiological parameters of 365 MB patients who were divided in two groups (with and without reactions) revealed that deterioration of nerve function was more frequent than improvement in both groups. Similar to the findings of Capadia et al., our results revealed high frequencies of poor outcomes for CSA (up to 25%) and echogenicity (45.2%) in the patients without reactions, sometimes with greater percentages of abnormalities than patients with reactions. These results show that leprosy neuropathy can deteriorate even in patients that received the recommended doses of the WHO-MDT and in patients without reactions.

The MB and the patients with reactions showed the opposite tendency; they presented reductions in CSA, ΔCSA and ΔUtpt for some nerves. Although we observed statistically significant reductions in the means of these measurements, the clinical significance is uncertain, as the magnitude of the reduction was small. We observed a higher frequency of poor CSA outcomes in MB compared to PB (p>0.05) and in patients with reactions compared to those without reactions (p<0.05), suggesting no significant improvement. Some of the patients of the group with reactions were still receiving anti-reaction treatment at the time of post-treatment US; therefore, it is possible that the presence of poor CSA outcomes were partially due to the persistence of the inflammatory process in this group. In addition, the reduction in nerve diameter is not necessarily a signal of improvement; it can represent the evolution to nerve fibrosis and atrophy. In the US exams, the fascicular changes associated with nerve inflammation, fibrosis and atrophy are represented by the echogenicity abnormalities [8–10,13,29]. We observed higher frequencies of echogenicity abnormalities in MB (p<0.05) and in patients with reactions (p>0.05), indicating that the reductions in nerve measurements might have been due to these processes.

The analyses of risk factors for poor US outcomes revealed that the odds for deterioration of anatomical changes detected by US seem to be only slightly higher in patients with reactions, whereas MB and PB patients had similar odds. Although previous studies have demonstrated that MB patients have a higher risk of developing or worsening nerve function impairment [24,25], the differences between our study design and previous studies preclude definite conclusions.

Intraneural or perineural Doppler detection is considered a marker of active neuritis [8,9,13,16,29] and our results confirm the association between Doppler signal and neuritis. We did not observe higher odds for having Doppler signal in the group with reactions and we detected a smaller frequency of Doppler signal after treatment (p>0.05). As all the patients with reactions received MDT and anti-reaction treatment, our results indicate that adequate treatment can diminish the acute inflammatory process caused by the bacillus and/or by immune reaction [8]. The detection of intraneural Doppler in one patient without clinical signs of reaction that developed active neuritis afterwards indicates that Doppler may detect subclinical reactions allowing for prompt treatment. Intraneural Doppler can be the first sign of nerve damage and it may have a role predicting reactions [8,9].

It has been demonstrated that patients with long standing disease can have important nerve echogenicity abnormalities without significant enlargement [8]. In our study we did not observe significant differences among the three groups for disease duration regarding nerve enlargement; however, we also did not observe differences in the frequencies of echogenicity abnormalities. We defined as long disease duration the presence of symptoms for more than 5 years. Martinoli et al. studied patients with disease durations ranging from 20 to 51 years; therefore, it is possible that the time frame defined as “long disease duration” in our study was not long enough to reveal differences between groups and future US exams (5 to 10 years after completion of WHO-MDT) should be done in these patients to investigate late nerve changes.

One major limitation of our study was the absence of correlation between nerve anatomical changes and nerve function abnormalities. Although neurophysiology studies could provide important information, we consider that our results can improve the understanding of the evolution of anatomic nerve changes detected by US. Similarly, the correlation between US findings and clinical symptoms and incapacity grade should be explored in future studies. Another limitation of the study was the relatively small sample size given that the standard deviation was great for some measurements. As the study was performed in a Leprosy Reference Center, we included a small number of PB patients, which can weaken the generalization of conclusions for this group. However, this limitation is observed in the majority of studies investigating leprosy neuropathy.

The most feared consequences of leprosy are due to nerve damage. Previous studies have shown that MDT does not stop the progression of nerve function impairment [5,17,26,27]. The present study shows that, similar to the results concerning nerve function, the anatomic nerve changes caused by leprosy may not improve significantly with treatment. Furthermore, they can worsen even in the PB and in the patients without reactions. US is an accurate method for detecting nerve enlargement, as was demonstrated by the high ICC values in our study and in previous studies [10,13,30]. In addition, it provides useful information about active inflammatory process (Doppler) and fascicular abnormalities (echogenicity) [8,9,11,16,13,14,29]. As the stigma related to leprosy is due to the consequences of neuropathy, it is essential the improvement of diagnostic and therapeutic procedures focusing on peripheral nerve involvement.

## Supporting Information

S1 Table Clinical data and pre- and post-treatment US findings of each patient included in the study. Legend: ID: patient identification; RJ: Modified Ridley-Jopling classification; Upt: ulnar nerve, proximal to the cubital tunnel; Ut: ulnar nerve at the cubital tunnel; I: indeterminate leprosy; TT: tuberculoid; BT: borderline-tuberculoid; BB: borderline-borderline; BL: borderline-lepromatous; LL: lepromatous; nl: normal; abn: abnormal; NP: measurement not performed (amputation, cutaneous ulcers or other cutaneous alterations at the site of examination).
(DOCX)

S1 Checklist STROBE Statement Checklist. Legend: checklist of items that should be included in reports of observational studies.
(PDF)

## References

[1] Rodrigues LC, Lockwood DNJ. Leprosy now: epidemiology, progress, challenges, and research gaps. Lancet Infect Dis 2011;11(6):464–470. 10.1016/S1473-3099(11)70006-8 21616456

[2] World Health Organization. (2009) WHO. Enhanced global strategy for further reducing the disease burden due to leprosy: 2011–2015. 20306634

[3] Lockwood DNJ, Saunderson PR. Nerve damage in leprosy: a continuing challenge to scientists, clinicians and service providers. Int Health. Royal Society of Tropical Medicine and Hygiene 2012;4(2):77–85. 10.1016/j.inhe.2011.09.006 24029146

[4] Wilder-Smith EP, Van Brakel WH. Nerve damage in leprosy and its management. Nat Clin Pract Neurol 2008;4(12):656–663. 10.1038/ncpneuro0941 19002133

[5] Leprosy as a neurological disease. Lancet Neurol 2009 3;8(3):217 10.1016/S1474-4422(09)70026-2 19233026

[6] Van Brakel WH, Saunderson PR, Shetty VP, Brandsma JW, Post E, Jellema R, et al International workshop on neuropathology in leprosy-consensus report. Lepr Rev 2007;78:416–433. 18309718

[7] Chen S, Wang Q, Tongsheng C, Ming Z. Inter-observer reliability in assessment of sensation of skin lesion and enlargement of peripheral nerves in leprosy patients. Lepr Rev 2006;77:371–376. 17343224

[8] Martinoli C, Derchi LE, Bertolotto M, Gandolfo N, Bianchi S, Fiallo P, et al US and MR imaging of peripheral nerves in leprosy. Skeletal Radiol 2000;29(3):142–150. 10794551 10.1007/s002560050584

[9] Jain S, Visser LH, Praveen TLN, Rao PN, Surekha T, Ellanti R, et al High-resolution sonography: a new technique to detect nerve damage in leprosy. PLoS Negl Trop Dis 2009; 3(8): e498 10.1371/journal.pntd.0000498 19668356 PMC2716078

[10] Elias J, Nogueira-Barbosa MH, Feltrin LT, Furini RB, Foss NT, Marques W. Role of Ulnar Nerve Sonography in Leprosy Neuropathy With Electrophysiologic Correlation. J Ultrassound Med 2009;28:1201–1209. 10.7863/jum.2009.28.9.1201 19710218

[11] Slim FJ, Faber WR, Maas M. The role of radiology in nerve function impairment and its musculoskeletal complications in leprosy. Lepr Rev 2009;80:373–387. 20306636

[12] Frade MAC, Nogueira-Barbosa MH, Lugão HB, Furini RB, Júnior WM, Foss NT. New sonographic measures of peripheral nerves : a tool for the diagnosis of peripheral nerve involvement in leprosy. Mem Inst Oswaldo Cruz 2013;108(3):257–262. 10.1590/S0074-02762013000300001 PMC4005566 23778664

[13] Goedee HS, Brekelmans GJF, van Asseldonk JTH, Beekman R, Mess WH, Visser LH. High resolution sonography in the evaluation of the peripheral nervous system in polyneuropathy—a review of the literature. Eur J Neurol 2013;20:1342–1351. 10.1111/ene.12182 23701599

[14] Polat Ekinci A, Karabacak E, Tekin L, Özarmağan G, Özçakar L. Ultrasound imaging for the follow-up of patients with leprosy: a pictorial essay. Br J Dermatol 2015;172(1):265–267. 10.1111/bjd.13421 25244462

[15] Lugão HB, Nogueira-Barbosa MH, Marques W Jr, Foss NT, Frade MAC. Asymmetric Nerve Enlargement : A Characteristic of Leprosy Neuropathy Demonstrated by Ultrasonography. PLoS Negl Trop Dis 2015;9(12): e0004276 10.1371/journal.pntd.0004276 26646143 PMC4672904

[16] Bathala L, Kumar K, Pathapati R, Jain S, Visser LH. Ulnar neuropathy in hansen disease: clinical, high-resolution ultrasound and electrophysiologic correlations. J Clin Neurophysiol 2012;29(2):190–193. 10.1097/WNP.0b013e31824d969c 22469686

[17] Capadia GD, Shetty VP, Khambati FA, Ghate SD. Effect of corticosteroid usage combined with multidrug therapy on nerve damage assessed using nerve conduction studies: a prospective cohort study of 365 untreated multibacillary leprosy patients. J Clin Neurophysiol 2010;27(1):38–47. 10.1097/WNP.0b013e3181cb426d 20087206

[18] Van Brakel WH, Nicholls PG, Das L, Barkataki P, Suneetha SK, Jadhav RS, et al The INFIR Cohort Study: investigating prediction, detection and pathogenesis of neuropathy and reactions in leprosy. Methods and baseline results of a cohort of multibacillary leprosy patients in north India. Lepr Rev 2005;76:14–34. 15881033

[19] Samant G, Shetty VP, Uplekar MW, Antia NH. Clinical and electrophysiological evaluation of nerve function impairment following cessation of multidrug therapy in leprosy. Lepr Rev 1999;70(1):10–20. 10405539 10.5935/0305-7518.19990005

[20] Ridley DS, Jopling WH. Classification of leprosy according to immunity. A five-group system. Int J Lepr Other Mycobact Dis 1966;34:255–273. 5950347

[21] World Health Organization. (1998) WHO. Expert Commitee on Leprosy: seventh report.

[22] Portney L, Watkins M. Foundations of Clinical Research Applications to Practice. Prentice Hall: New Jersey 2000.

[23] Albert A and Anderson JA. On the Existence of Maximum Likelihood Estimates in Logistic Regression Models. Biometrika 1984; 71:1–10. 10.1093/biomet/71.1.1

[24] Croft RP, Nicholls PG, Steyerberg EW, Richardus JH, Cairns W, Smith S. A clinical prediction rule for nerve-function impairment in leprosy patients. Lancet 2000;355:1603–1606. 10821364 10.1016/s0140-6736(00)02216-9

[25] Schuring RP, Richardus JH, Steyerberg EW, Pahan D, Faber WR, Oskam L. Preventing nerve function impairment in leprosy: validation and updating of a prediction rule. PLoS Negl Trop Dis 2008 1;2(8): e283 10.1371/journal.pntd.0000283 18846229 PMC2565693

[26] Van Veen NHJ, Nicholls PG, Smith WCS, Richardus JH. Corticosteroids for treating nerve damage in leprosy. Cochrane database Syst Rev 2007;(2):CD005491. 10.1002/14651858.CD005491.pub2 17443594

[27] Van Brakel WH, Nicholls PG, Wilder-Smith EP, Das L, Barkataki P, Lockwood DNJ. Early diagnosis of neuropathy in leprosy-comparing diagnostic tests in a large prospective study (the INFIR cohort study). PLoS Negl Trop Dis 2008 1;2(4): e212 10.1371/journal.pntd.0000212 18382604 PMC2270341

[28] Visser LH, Jain S, Lokesh B, Suneetha S, Subbanna J. Morphological changes of the epineurium in leprosy: a new finding detected by high-resolution sonography. Muscle Nerve 2012;46(1):38–41. 10.1002/mus.23269 22644782

[29] Jain S, Visser LH, Yerasu MR, Raju R, Meena AK, Lokesh B, et al Use of high resolution ultrasonography as an additional tool in the diagnosis of primary neuritic leprosy: a case report. Lepr Rev 2013;84:161–165. 24171244

[30] Cartwright MS, Passmore L V, Yoon J-S, Brown ME, Caress JB, Walker FO. Cross-sectional area reference values for nerve ultrasonography. Muscle Nerve 2008;37(5):566–571. 10.1002/mus.21009 18351581

