# Supplementary material for: Ultrasonography of Leprosy Neuropathy: A Longitudinal Prospective Study
Source: PLoS Negl Trop Dis. 2016 Nov 16;10(11):e0005111. doi: 10.1371/journal.pntd.0005111 (PMC5112942; doi:10.1371/journal.pntd.0005111)
Supplement: S1 Table — Legend: ID: patient identification; RJ: Modified Ridley-Jopling classification; Upt: ulnar nerve, proximal to the cubital tunnel; Ut: ulnar nerve at the cubital tunnel; I: indeterminate leprosy; TT: tuberculoid; BT: borderline-tuberculoid; BB: borderline-borderline; BL: borderline-lepromatous; LL: lepromatous; nl: normal; abn: abnormal; NP: measurement not performed (amputation, cutaneous ulcers or other cutaneous alterations at the site of examination). (DOCX) [file pntd.0005111.s001.docx]

**S1 Table. Clinical data and pre- and post-treatment US findings of each patient included in the study.**

| Clinical data | | | | Pre-treatment US | | | | | | | | | | Post-treatment US | | | | | | | | | |
| --- | --- | --- | --- | --- | --- | --- | --- | --- | --- | --- | --- | --- | --- | --- | --- | --- | --- | --- | --- | --- | --- | --- | --- |
| ID | RJ | Cutaneous reactions (types 1 or 2) | neuritis (yes / no) | Right Upt CSA (mm²) | Right Ut CSA (mm²) | Right M CSA (mm²) | Right CF CSA (mm²) | Left Upt CSA (mm²) | Left Ut CSA (mm²) | Left M CSA (mm²) | Left CF CSA (mm²) | Ecogenicity | Doppler | Right Upt CSA (mm²) | Right Ut CSA (mm²) | Right M CSA (mm²) | Right CF CSA (mm²) | Left Upt CSA (mm²) | Left Ut CSA (mm²) | Left M CSA (mm²) | Left CF CSA (mm²) | Ecogenicity | Doppler |
| 1 | BB | no | yes | 12.8 | 14 | 6.3 | 12.7 | 10.5 | 7.9 | 6.3 | 10.1 | nl | nl | 6 | 14 | 6 | 13 | 6 | 4 | 4 | 12 | abn | nl |
| 2 | BT | no | no | 5 | 7 | 6 | 23 | 8 | 6 | 5 | 19 | nl | abn | 4 | 7 | 6 | 13 | 5 | 8 | 5 | 15 | nl | nl |
| 3 | BB | 1 | no | 15 | 24 | 8 | 11 | 7 | 8 | 8 | 7 | abn | nl | 20.6 | 20 | 5.1 | 22.2 | 4.3 | 5.6 | 7.2 | 11.9 | abn | nl |
| 4 | BT | no | no | 7 | 8 | 7 | 13 | 7 | 7 | 10 | 16 | nl | nl | 7 | 6 | 7 | 14 | 5 | 9 | 10 | 17 | nl | nl |
| 5 | BL | 1 | no | 8 | 7.7 | 7.3 | 15.2 | 6.7 | 7.8 | 6.4 | 16.9 | nl | nl | 5.6 | 9.7 | 5.4 | 16.6 | 6.7 | 8.6 | 7.4 | 20.2 | nl | nl |
| 6 | BL | 1 | yes | 7.2 | 6.5 | 5.4 | 13.8 | 18.1 | 11.2 | 6.2 | 14 | nl | abn | 5 | 7.4 | 14.9 | 13.2 | 9.4 | 8.5 | 8.4 | 14.9 | abn | nl |
| 7 | BB | no | no | 7 | 12.5 | 6.5 | 14.7 | 6.5 | 15.8 | 9.2 | 15.6 | abn | nl | 8 | 11 | 7 | 13 | 7 | 13 | 7 | 13 | abn | nl |
| 8 | BL | no | no | 9.8 | 6.6 | 15.9 | 25.3 | 10.3 | 8.6 | 14.1 | 16.9 | nl | nl | 6.5 | 5 | 9.3 | 14 | 8 | 6.2 | 5 | 19 | nl | nl |
| 9 | BT | no | yes | 6.7 | 15.8 | 8 | 13.4 | NP | 112 | 10.8 | 13 | abn | nl | 6.9 | 6 | 7.4 | 9.6 | 148 | 47.7 | 6.6 | 8.9 | abn | nl |
| 10 | LL | 2 | no | 9.2 | 17.9 | 8.9 | 12 | 6 | 12 | 9 | 11 | nl | nl | 16 | 5 | 12 | 7 | 17 | 3 | 12 | 11 | abn | nl |
| 11 | LL | 2 | no | 8.9 | 10.1 | 8.4 | 19.3 | 9.7 | 8.8 | 8.8 | 20.2 | nl | nl | 11.6 | 11.5 | 9.2 | 12.2 | 10.5 | 8.7 | 10.1 | 17.1 | nl | nl |
| 12 | BB | 1 | no | 9.4 | 14.1 | 17.4 | 19.8 | 17.7 | 10.7 | 23.8 | 27.2 | abn | abn | 6.3 | 9.9 | 13.5 | 15.5 | 14.2 | 8 | 16.2 | 18.9 | nl | nl |
| 13 | BT | no | no | 8.1 | 11.1 | 9.7 | 21.3 | 8.6 | 12.7 | 7.1 | 9.8 | abn | nl | 6.7 | 12.6 | 13.6 | 23.3 | 9.3 | 12.7 | 11.6 | 23.5 | abn | nl |
| 14 | BT | no | no | 6.1 | 11 | 6.8 | 11.9 | 8.8 | 10.2 | 5.5 | 10.8 | nl | nl | 6 | 13 | 7 | 20 | 7 | 11 | 7 | 16 | abn | nl |
| 15 | TT | no | no | 6.9 | 6 | 5.3 | 11.2 | 5.2 | 7.2 | 6.8 | 10.6 | nl | nl | 6.1 | 7 | 7.1 | 11.8 | 5.5 | 6.9 | 6.3 | 12.7 | nl | nl |
| 16 | BB | no | yes | 4.4 | 8.1 | 5.8 | 9.3 | 4.6 | 7.4 | 6.9 | 11 | nl | nl | 7 | 8 | 7 | 16 | 7 | 7 | 6 | 15 | nl | nl |
| 17 | BL | 1 | no | 4 | 8 | 7 | 11 | 5 | 7 | 5 | 9 | nl | nl | 7.1 | 12.7 | 6.6 | 23 | 7.8 | 9.5 | 7.6 | 21 | nl | nl |
| 18 | BB | no | no | 3 | 6 | 5 | 9 | 4 | 5 | 5 | 12 | nl | nl | 5 | 4 | 5 | 16 | 7 | 5 | 5 | 12 | nl | nl |
| 19 | I | no | no | 6 | 5 | 3 | 5 | 6 | 4 | 6 | 6 | nl | nl | 5 | 3 | 2 | 6 | 3 | 5 | 4 | 8 | nl | nl |
| 20 | LL | 2 | yes | 16 | 10 | 7 | 14 | 40 | 14 | 8 | 16 | abn | abn | 18 | 12 | 8 | 17 | 46 | 15 | 8 | 14 | abn | nl |
| 21 | TT | no | yes | 10 | 5 | 6 | 5 | 6 | 6 | 7 | 5 | nl | nl | 7 | 6 | 6 | 14 | 5 | 8 | 6 | 18 | nl | nl |
| 22 | BT | no | no | 2.8 | 4 | 2.6 | 2.6 | 3.3 | 4.1 | 2.8 | 2.7 | nl | nl | 5.9 | 6.8 | 6.7 | 7.9 | 5.3 | 5.7 | 4.5 | 4 | nl | nl |
| 23 | BL | 1 | no | 8.8 | 10.4 | 21.5 | 20.5 | 14.3 | 17 | 20 | 25 | nl | nl | 7 | 9 | 18 | 20 | 8 | 15 | 14 | 17 | nl | nl |
| 24 | BL | 1 | yes | 19.8 | 11.5 | 23.5 | 22.2 | 21.3 | 13.4 | 23.2 | 40.2 | abn | nl | 13 | 11 | 18 | 26 | 16 | 13 | 20 | 27 | abn | nl |
| 25 | BT | no | yes | 8 | 9 | 11 | 40 | 7 | 8 | 12 | 50 | nl | nl | 5.9 | 8.7 | 15 | 42 | 7.8 | 7.5 | 15 | 43 | abn | nl |
| 26 | BT | no | no | 5 | 11 | 6 | 24 | 10 | 9 | 10 | 20 | abn | nl | 10.9 | 13.2 | 9.4 | 32.7 | 9.5 | 8.6 | 8.7 | 20.5 | abn | nl |
| 27 | BT | no | yes | NP | 27 | 11 | 12 | NP | 29 | 9 | 14 | abn | nl | 8 | 24 | 8 | 25 | 8 | 23 | 8 | 20 | abn | nl |
| 28 | TT | no | yes | 8 | 13 | 8 | 10 | 12 | 7 | 7 | 10 | nl | nl | 7 | 7 | 6 | 17 | 9 | 8 | 7 | 22 | nl | nl |
| 29 | BB | no | no | 7 | 34 | 8 | 9 | 4 | 7 | 8 | 9 | nl | nl | 27 | 14 | 6 | 10 | 7 | 5 | 7 | 9 | abn | nl |
| 30 | BT | no | yes | 12.5 | 8.5 | 7.7 | 20.5 | 10.2 | 7.7 | 6.9 | 27.6 | abn | nl | 6.7 | 6.5 | 6 | 13.9 | 7.3 | 7.8 | 7.4 | 16.9 | abn | nl |
| 31 | BT | no | no | 6 | 21.7 | 6.1 | 16.9 | 6.5 | 25.1 | 5.9 | 27.9 | abn | nl | 5.2 | 18.8 | 5.3 | 20.4 | 5.7 | 21.3 | 5.1 | 28 | abn | nl |
| 32 | BB | no | yes | 8 | 9 | NP | NP | 6 | 7 | NP | NP | nl | nl | 7 | 5.8 | 7.1 | 8.2 | 5.3 | 6 | 5.1 | 11 | nl | nl |
| 33 | BT | 1 | no | 8 | 9 | 9 | 15 | 8 | 7 | 11 | 16 | nl | nl | 8 | 7 | 6 | 18 | 7 | 7 | 6 | 17 | nl | nl |
| 34 | BT | 1 | no | 7.1 | 8.6 | 11.1 | 12.4 | 57 | 15.2 | 10.9 | 11.6 | abn | abn | 7.2 | 9.6 | 7.3 | 12.3 | 32.3 | 9 | 7.7 | 14.1 | abn | nl |
| 35 | TT | no | no | 5.3 | 6.1 | 5.4 | 9.6 | 10.8 | 5.6 | 5.4 | 9.2 | nl | nl | 4.5 | 7.1 | 5.6 | 14 | 7 | 7.1 | 7.1 | 16.8 | nl | nl |
| 36 | BT | no | yes | 4.4 | 5.8 | 5.3 | 10.8 | 28.1 | 7 | 4.7 | 9.3 | abn | nl | NP | 5.5 | 8 | 12 | 30 | 7 | 4.7 | 9 | abn | abn |
| 37 | BL | 1 and 2 | no | 7.4 | 6 | 8.5 | 27.9 | 6.7 | 7.6 | 6.3 | 21 | nl | nl | 5.8 | 6.5 | 5.1 | 14.2 | 5.8 | 6.5 | 6.5 | 16.7 | nl | nl |
| 38 | BT | no | no | 6.2 | 6.5 | 7 | 19 | 7.7 | 6.1 | 6.1 | 21 | nl | abn | 6 | 6 | 9 | 18 | 7 | 8 | 8 | 16 | nl | nl |
| 39 | BT | no | no | 7.2 | NP | 4.2 | 11.9 | 13.6 | 7.7 | 17.6 | 45.2 | abn | abn | 5 | 8 | 5 | 10 | 4 | 6 | 10 | 28 | abn | nl |
| 40 | BB | 1 | yes | 22 | 23.9 | 9.5 | 12.8 | 6.3 | 7.4 | 7.4 | 10.9 | abn | nl | 27.2 | 24.6 | 23.7 | 8.8 | 5.2 | 7.9 | 7.2 | 11.4 | abn | abn |
| 41 | BT | no | yes | 8 | 6 | 6 | 15 | 9 | 6 | 6 | 13 | abn | nl | 8 | 7 | 7 | 10 | 5 | 7 | 7 | 10 | abn | nl |
| 42 | BB | 1 | no | 8.4 | 10.9 | 5.4 | 11.9 | 8 | 8.5 | 6.8 | 12.1 | nl | nl | 7 | 9 | 17 | 21 | 9 | 9 | 9 | 21 | abn | nl |
| 43 | I | no | no | 7.8 | 9.5 | 7.6 | 8.2 | 8.4 | 8 | 5.2 | 12 | nl | nl | 8.4 | 6.4 | 6.9 | 8.3 | 7.3 | 7.6 | 5 | 13.8 | nl | nl |
| 44 | BB | no | no | 4.7 | 8.9 | 4.7 | 8.2 | 4.2 | 5.1 | 6.2 | 12.2 | nl | nl | 4 | 7 | 5 | 14 | 5 | 6 | 5 | 18 | abn | nl |
| 45 | TT | no | yes | 7 | 8 | NP | 10.4 | 6.8 | 8.8 | NP | 63.1 | abn | nl | 7 | 8 | 6.6 | 14.1 | 5.8 | 7.1 | 6 | 78.1 | abn | nl |
| 46 | BB | no | yes | 16 | 13 | 21 | 28 | 28 | 18 | 9 | 25 | nl | nl | 17 | 15 | 16 | 26 | 23 | 15 | 9 | 21 | abn | abn |
| 47 | BB | no | yes | 4 | 3.4 | 4.9 | 8.8 | 3.9 | 4.7 | 5.1 | 5.7 | nl | nl | 4 | 3 | 3 | 9 | 3 | 3 | 3 | 10 | nl | nl |
| 48 | BB | no | yes | 2.9 | 5.6 | 4.5 | 29.5 | 8.2 | 6.2 | 6.4 | 37 | nl | nl | 6 | 6 | 12 | 30 | 7 | 9 | 13 | 35 | nl | nl |
| 49 | BB | no | yes | 9.7 | 14.8 | 9.4 | 21.2 | 10.6 | 9.9 | 20 | 21.5 | nl | nl | 9.9 | 9.9 | 7.3 | 22.1 | 7.9 | 7.2 | 19.2 | 25 | abn | nl |
| 50 | BL | no | no | 4.8 | 5.8 | 7.7 | 46 | 4.7 | 5.4 | 8.7 | 15 | abn | abn | 6 | 7 | 6 | 27 | 4 | 6 | 9 | 12 | abn | nl |
| 51 | BB | no | no | 6 | 8 | 6 | 11 | 8 | 8 | 5 | 12 | nl | nl | 7.3 | 7.7 | 4 | 9.9 | 5.8 | 8.7 | 5.3 | 11.1 | nl | nl |
| 52 | BB | no | no | 10 | 12 | 8 | 45 | 12 | 15 | 9 | 25 | abn | nl | 10 | 12 | 12.1 | 24.3 | 12.8 | 13.4 | 13.7 | 19.9 | abn | nl |
| 53 | TT | 1 | no | 5.3 | 5.2 | 6.5 | 23 | 6 | 6.8 | 5.4 | 30 | nl | nl | 5.9 | 7.8 | 7.2 | 25.7 | 6.5 | 6 | 5.7 | 31.7 | abn | nl |
| 54 | BB | no | no | 8 | 8 | 9.2 | 12.9 | 6.6 | 8.1 | NP | 18 | nl | nl | 5.4 | 6.5 | 7.3 | 12.9 | NP | NP | 7 | 16.5 | nl | nl |
| 55 | BB | no | no | 5 | 13.1 | 12.4 | 8.2 | 6.5 | 9.1 | 10 | 13 | nl | nl | 5.5 | 9.7 | 8.9 | 12.9 | 4.5 | 10.1 | 7 | 15.6 | nl | nl |
| 56 | BT | no | yes | 6.8 | 7 | 8 | 14.1 | 5.7 | 5.6 | 6.5 | 15.3 | nl | nl | 4 | 5 | 4 | 14 | 4 | 5 | 5 | 12 | nl | nl |
| 57 | BL | 1 | no | 5.9 | 6.2 | 10.7 | 13 | 11.8 | 12 | 20.1 | 12.1 | abn | abn | 4 | 5.4 | 15.2 | 16.3 | 11.8 | 11 | 15.7 | 15.3 | abn | nl |
| 58 | BT | no | yes | 5 | 6 | 6 | 8 | 12 | 26 | 6 | 8 | abn | nl | 6.5 | 4.2 | 5.7 | 12.2 | 12.1 | 14.9 | 9 | 10.8 | abn | nl |
| 59 | BB | no | no | 7 | 8 | 7.2 | 11.5 | 5.1 | 8.1 | 6.8 | 10.7 | abn | nl | 5.9 | 6.8 | 7.9 | 13.3 | 5.9 | 7.8 | 6.1 | 13.2 | nl | nl |
| 60 | BB | no | no | 5 | 5 | 8 | 15 | 5 | 6 | 8 | 15 | nl | nl | 8.4 | 5.6 | 9.4 | 10.9 | 5.5 | 6.2 | 8 | 18.6 | nl | nl |
| 61 | I | no | no | 5.3 | 4.6 | 7.1 | 7.2 | 6.1 | 7.1 | 7.6 | 11 | nl | nl | 6.7 | 4.9 | 7.4 | 14.9 | 6.7 | 5.4 | 5.8 | 16.1 | nl | nl |
| 62 | BB | 1 | no | 6.7 | 17.6 | 6.5 | 34.9 | 6.9 | 12.3 | 6.8 | 40.9 | abn | abn | 18 | 12 | 24 | 32 | 20 | 6 | 9 | 29 | abn | nl |
| 63 | BB | 1 | no | 29.5 | 22.4 | 5.6 | 10.8 | 47.9 | 35.7 | 10.2 | 9.6 | abn | nl | 29.5 | 16 | 10 | 20 | 30.5 | 28.5 | 16 | 15 | abn | nl |
| 64 | BT | no | no | 6.5 | 23.8 | 27.7 | 27.2 | 8.4 | 30.6 | 27.5 | 15.5 | abn | abn | 11.3 | 20.1 | 18.4 | 27.3 | 13.3 | 26.7 | 22.1 | 29 | abn | nl |
| 65 | LL | no | no | 19 | 15.4 | 12.5 | 24 | 18.8 | 11.4 | 17.7 | 21.2 | abn | nl | 15 | 20 | 7 | 18 | 15 | 10 | 15 | 16 | abn | nl |
| 66 | BB | no | no | 8.9 | 12 | 9 | 13 | 7.8 | 13 | 9 | 13 | abn | nl | 7 | 9 | 8 | 8 | 8 | 10 | 9 | 7 | abn | abn |
| 67 | BB | 1 | yes | 6 | 7 | 7 | 17 | 9 | 14 | 25 | 14 | abn | abn | 5 | 10 | 8 | 23 | 13 | 19 | 25 | 15 | abn | abn |
| 68 | BL | no | yes | 17 | 14 | 19 | 23 | 17 | 14 | 18 | 30 | abn | abn | 16.4 | 10.6 | 15.2 | 11.8 | 18.8 | 9.8 | 16 | 15.3 | abn | nl |
| 69 | BB | no | no | 3.7 | 7.5 | 4.2 | 16.8 | 4.9 | 7.8 | 5 | 13.8 | nl | nl | 3.2 | 3.6 | 4 | 10.4 | 3.5 | 5.2 | 5.4 | 14.6 | nl | nl |
| 70 | BB | no | yes | 6 | 7 | 6 | 24.2 | 5 | 6 | 6 | 29.5 | nl | nl | 7 | 7 | 8 | 29 | 7 | 9 | 8 | 24 | nl | abn |
| 71 | BB | no | no | 9 | 12 | 12 | 23 | 10 | 15 | 13 | 31 | abn | nl | 15 | 11 | 9 | 32 | 11 | 17 | 11 | 33 | abn | nl |
| 72 | BT | 1 | yes | 6 | 8 | 6 | 13 | 6 | 9 | 7 | 15 | abn | abn | 7.1 | 4.3 | 6.8 | 8.9 | 10.8 | 7.9 | 5.4 | 15.4 | abn | nl |
| 73 | BB | no | yes | 4.8 | 7.7 | 4.9 | 11.3 | 4.1 | 7.8 | 5.3 | 12.9 | nl | nl | 4 | 4 | 7 | 7 | 3 | 6 | 6 | 6 | nl | nl |

Legend: ID: patient identification; RJ: Modified Ridley-Jopling classification; Upt: ulnar nerve, proximal to the cubital tunnel; Ut: ulnar nerve at the cubital tunnel; I: indeterminate leprosy; TT: tuberculoid; BT: borderline-tuberculoid; BB: borderline-borderline; BL: borderline-lepromatous; LL: lepromatous; nl: normal; abn: abnormal; NP: measurement not performed (amputation, cutaneous ulcers or other cutaneous alterations at the site of examination).
